# MEF2A nuclear translocation mediated by ROCK phosphorylation promotes EZH2 expression and peritoneal fibrosis

**DOI:** 10.1080/0886022X.2026.2664318

**Published:** 2026-05-17

**Authors:** Dandan Chen, Zhikang Sun, Ying Xu, Qinglian Wang

**Affiliations:** a Department of Nephrology, Xiangya Hospital Central South University, Changsha, Hunan, China; b Shandong Provincial Hospital, Shandong First Medical University, Jinan, Shandong, China

**Keywords:** Peritoneal fibrosis (PF), EZH2, MEF2A, ROCK, fasudil

## Abstract

Prolonged peritoneal dialysis (PD) frequently leads to peritoneal fibrosis (PF). This study aimed to define the role of the Rho-associated coiled-coil containing protein kinase (ROCK) signaling pathway and its downstream effectors, MEF2A and EZH2, in PF. *In vivo*, a rat PD model was treated with the ROCK inhibitor fasudil or adeno-associated virus-mediated MEF2A knockdown. *In vitro*, high glucose-stimulated human peritoneal mesothelial cells (HPMCs) were subjected to MEF2A knockdown, EZH2 overexpression, or fasudil treatment. Exposure of HPMCs to high glucose markedly upregulated total and phosphorylated MEF2A (p-MEF2A) and promoted its nuclear translocation. MEF2A knockdown suppressed high glucose-induced upregulation of EZH2 and epithelial-mesenchymal transition (EMT), whereas EZH2 overexpression reversed this protective effect. Dual-luciferase reporter and ChIP-qPCR assays confirmed that MEF2A binds to the EZH2 promoter and activates its transcription. Inhibition of the ROCK pathway with fasudil reversed the high glucose-induced upregulation of MEF2A and suppressed its phosphorylation and nuclear translocation. *In vivo*, MEF2A knockdown alleviated PF in rats undergoing to long-term PD. Consistently, oral fasudil treatment inhibited the dialysis-induced upregulation of MEF2A and EMT markers. These results reveal a novel ROCK/MEF2A/EZH2 axis in PF pathogenesis, highlighting MEF2A as a potential therapeutic target.

## 1. Introduction

Peritoneal dialysis (PD) is one of the most important renal replacement treatments in patients with end-stage renal disease [1,2]. However, prolonged treatment with high-glucose dialysate can initiate a cascade of events, beginning with structural and functional changes in peritoneal mesothelial cells, followed by peritoneal inflammation and neovascularization, and eventually resulting in peritoneal fibrosis (PF) [3,4]. Peritoneal fibrosis-induced hypofunction and ultrafiltration failure consequently compromise the long-term efficacy of PD and adversely impact patient prognosis [5,6]. However, the pathogenesis of PF remains incompletely understood. This study therefore aims to elucidate its underlying mechanisms.

Epithelial-mesenchymal transition (EMT) occurs during the process of peritoneal fibrosis [7]. In this process, peritoneal mesothelial cells undergo a phenotypic shift, transforming from epithelial to mesenchymal states. This transformation is characterized by distinct morphological and functional alterations: the cell morphology changes from cobblestone to slender spindle, accompanied by the loss of cell polarity, cytoskeleton changes, and the ability of cells to acquire invasion and movement, etc., which can be manifested by the absence of epithelial cell markers (such as cytokeratins and E-cadherin), the expression of mesenchymal cell markers (e.g., N-cadherin, Vimentin and α-SMA)[8,9]. These phenotypic changes reduce intercellular adhesion and enhanced migration movement, thus participating in peritoneal fibrosis.

The Rho-associated coiled-coil containing protein kinase (ROCK) signaling pathway is involved in inducing cytoskeleton reorganization, cell migration and stress fiber formation, related to various physiological functions such as endothelial permeability, tissue contraction and growth, and participates in the occurrence of diabetic nephropathy, tumor and other diseases [10–12]. This pathway has attracted increasing attention as a target for drug development. Rho can promote the formation and elongation of stress fibers, actin contraction and directional adhesion. Rho has two states: GDP binding inactivation and GTP binding activation, and the relative ratio of the two states is regulated by GTPase-activating proteins (GAPs) and guanine nucleotide exchange factors (GEFs)[13]. In its active form, Rho binds to and activates ROCK. Activated ROCK then phosphorylates downstream substrates to drive cytoskeletal remodeling, including inducing actin filament stabilization, facilitating actomyosin contraction, and regulate microtubule dynamics [14,15]. Moreover, in previous studies, the ROCK signaling pathway has been demonstrated to be involved in multiple fibrotic diseases [16,17]. Although the ROCK signaling pathway has been demonstrated to be involved in peritoneal fibrosis [18,19], its key downstream effectors and precise mechanistic role remain poorly defined. This study therefore aimed to delineate the downstream targets of this pathway and elucidate the specific molecular mechanisms through which it drives fibrotic progression.

The MEF2 family of myocyte enhancer factors has four members: MEF2A, MEF2B, MEF2C, and MEF2D. Activation of MEF2 transcription factors *in vivo* can promote the hypertrophy of cardiac muscle and skeletal muscle through calcium-dependent signal transduction pathways [20]. It has been found that MEF2A plays a huge role in the development of fibrosis of hepatic stellate cells and cardiomyocytes [21,22]. Moreover, Xueying Chen et al. demonstrated that inhibition of MEF2A could partially improve diabetic cardiac fibrosis by regulating EMT [23]. Therefore, we speculate that MEF2A also plays a partial role in peritoneal fibrosis, which has been demonstrated in this study. Additionally, we aimed to elucidate the specific mechanism by which MEF2A plays a role in the process of peritoneal fibrosis.

Enhancer of zeste homolog 2 (EZH2) is the enzymatic subunit of the polycomb repressive complex 2 (PRC2), a histone methyltransferase that trimethylates lysine (H3K27me3) at position 27 of histone H3 to promote transcriptional silencing and inhibit the expression of target genes [24]. Most of these target genes are involved in inhibiting cell proliferation and promoting cell differentiation, and the overexpression or overenhanced function of EZH2 will enable the excessive silencing of the above gene expression, ultimately leading to the disorder of stem cell multiple differentiation potential and tumorigenesis [25,26]. EZH2 is highly expressed in many human cancers and promotes cancer development and malignancy, such as breast cancer [27], prostate cancer [28], gastric cancer [29], etc. It has been found that EZH2 also plays an important role in the process of peritoneal fibrosis [30], but the specific mechanism is still what we need to further explore at present.

Through a series of *in vivo* and *in vitro* experiments, we found that the amount of MEF2A and EZH2 expression was reduced after the administration of ROCK inhibitor under high glucose stimulation, and the expression of EZH2 was also decreased after the knockdown of MEF2A.This proves that under high glucose stimulation, the ROCK pathway is activated, which increases the downstream MEF2A expression level, which then affects the expression of EZH2 and ultimately promotes the development of peritoneal fibrosis. This study provides novel insights into the pathogenesis of peritoneal fibrosis.

## 2. Materials and methods

### 2.1. Cell culture and treatment

The human peritoneal mesothelial cell line HMrSV5 was kindly provided by Professor Xueqing Yu (The First Affiliated Hospital, Sun Yat-sen University). Cells were maintained in DMEM/F12 medium supplemented with 10% fetal bovine serum (FBS) at 37 °C in a humidified atmosphere containing 5% CO_2_ and 95% air. Upon reaching confluence, the cells were detached using EDTA and subcultured. For experimental purposes, HMrSV5 cells were grown either in standard DMEM/F12 or in high-glucose DMEM/F12 medium containing 2.5% glucose. Transfection was performed when cell confluence reached 60–70%, and transfection efficiency was evaluated by Western blot analysis.

### 2.2. Cell transfection

The EZH2 overexpression plasmid was obtained from Genomeditech (Shanghai, China). The MEF2A siRNA was sourced from Hopson Biology (Shanghai, China). All aliquots were stored at −20 °C to ensure stability prior to use.

The primary antibody: anti-α-SMA (Proteintech, China), anti-Vimentin (Proteintech, China), anti-E-cadherin (Proteintech, China), anti-EZH2 (Abcam, UK), anti-MEF2A (BOSTER, China), anti-phospho-MEF2A (Bioss, China), anti-ROCK (Abcam, UK), anti-GAPDH (Proteintech, China). The horseradish peroxidase (HRP)-coupled goat anti-rabbit antibody and goat anti-mouse antibody were purchased from Proteintoch.

### 2.3. Western blot analysis

Protein extraction was performed by lysing cells with RIPA buffer supplemented with protease and phosphatase inhibitors on ice for 30 min. The lysates were then centrifuged at 12,000 × g for 30 min at 4 °C to collect the supernatant. Subsequently, the supernatant was mixed with 4× loading buffer, boiled at 100 °C for 10 min, and subjected to SDS-PAGE on 10% or 12% gels. The separated proteins were electrophoretically transferred onto PVDF membranes (Yarase, China). After blocking with 5% skim milk for 1 h at room temperature, the membranes were incubated overnight at 4 °C with the following primary antibodies: anti-GAPDH (PTG, 1:5000), anti-α-SMA (PTG, 1:2500), anti-Vimentin (CST, 1:1000), anti-E-cadherin (1:10000), anti-EZH2 (PTG, 1:2000), anti-MEF2A (PTG, 1:1000), anti-p-MEF2A (Boosen, 1:1000), and anti-ROCK (PTG, 1:1000). The following day, the membranes were incubated with horseradish peroxidase (HRP)-conjugated secondary antibodies at room temperature. Protein bands were visualized and quantified using ImageJ software.

### 2.4. Dual-luciferase reporter assay

The dual-luciferase reporter assay was conducted to validate the predicted MEF2A binding site on the EZH2 promoter, which was identified *via* the JASPAR database. Accordingly, a wild-type H-EZH2 promoter-luciferase reporter plasmid and two mutant versions were constructed (Genomeditech, Shanghai, China). 293 T cells, seeded in 6-well plates, were co-transfected at approximately 70% confluence with the MEF2A overexpression plasmid and one of the following: the wild-type reporter, a mutant reporter, or a negative control (NC) plasmid. Following a 48-h incubation, luciferase activity was quantified using the Dual-Luciferase^®^ Reporter Assay System (Promega, Madison, WI, USA) in strict accordance with the manufacturer’s guidelines.

### 2.5. Cellular immunofluorescence

HPMCs were blocked with goat serum for 30 min at room temperature to prevent nonspecific immunoreactivity. After being washed three times with PBS, the cells were incubated overnight at 4 °C with the following primary antibodies: anti-α-SMA (1:500), anti-EZH2 (1:250), anti-MEF2A (1:200), and anti-p-MEF2A (1:200). The following day, the cells were probed with either goat anti-rabbit IgG or goat anti-mouse IgG secondary antibodies for 1 h at room temperature. Following PBS washes, the cells were mounted with anti-fade mounting medium containing DAPI. Fluorescence images were acquired using a fluorescence microscope.

### 2.6. Histology and immunohistochemical staining

Paraffin-embedded tissues were sectioned at a thickness of 3 μm. After routine deparaffinization and hydration, sections were stained with hematoxylin and eosin (H&E) or Masson’s trichrome for histopathological evaluation. For immunohistochemistry, antigen retrieval was performed using citrate buffer (pH 6.0). Subsequently, endogenous peroxidases were blocked by incubation with 3% H_2_O_2_ for 10 min at room temperature. Following three washes with PBS, nonspecific binding sites were blocked with goat serum (10 min, room temperature). The sections were then incubated overnight at 4 °C with primary antibodies against α-SMA (1:200), Vimentin (1:200), and EZH2 (1:200), and MEF2A (1:200). After washing, the sections were incubated with a horseradish peroxidase (HRP)-conjugated secondary antibody for 30 min at room temperature. Signal was developed using diaminobenzidine (DAB) substrate, followed by counterstaining with hematoxylin. Finally, the sections were dehydrated, mounted with neutral resin, and imaged under a light microscope. Positive immunoreactivity was identified as a brown precipitate.

### 2.7. Animal experiments

All animal experiments were approved by the Animal Ethics Committee of Shandong Provincial Hospital Affiliated to Shandong First Medical University (Approval No. 2022-143). The study was performed on 6- to 8-week-old male Sprague-Dawley (SD) rats in accordance with the approved guidelines. The rats were divided into five experimental groups (*n* = 8 per group) as follows: a control group, a PDF group, a PDF group treated with AAV (PDF+ AAV), a PDF group treated with a negative control (PDF+NC), and a PDF group treated with Fasudil (PDF+fasudil). The PDF group received a daily intraperitoneal injection of 4.25% peritoneal dialysis solution (100 mL/kg) for 28 days. In contrast, the control group was given an equivalent daily volume of physiological saline.Three days before the PDF modeling, rats in the PDF + NC and PDF + AAV groups were pretreated with an intraperitoneal injection of the corresponding adeno-associated virus (AAV) or NC vectors. Subsequently, all rats in these groups received a daily intraperitoneal infusion of 4.25% peritoneal dialysis fluid at 100 mL/kg for 28 days. The PDF+Fasudil group was treated with daily intraperitoneal injections of peritoneal dialysis fluid in combination with continuous oral administration of fasudil (30 mg/kg). After the 28-day treatment period, all rats were anesthetized and euthanized by intraperitoneal injection of pentobarbital sodium (150 mg/kg). Subsequently, peritoneal tissues were collected for further experimental analysis.

### 2.8. Statistical analysis

All data are expressed as the mean ± standard deviation (SD) from at least three independent experiments. Statistical comparisons among groups were performed by one-way analysis of variance (ANOVA) followed by Dunnett’s post-hoc test. A *p*-value < 0.05 was considered statistically significant.

## 3. Results

### 3.1. High glucose upregulated the expression of MEF2A and p-MEF2A and promoted the nuclear translocation of p-MEF2A in HPMCs

**Figure 1.**
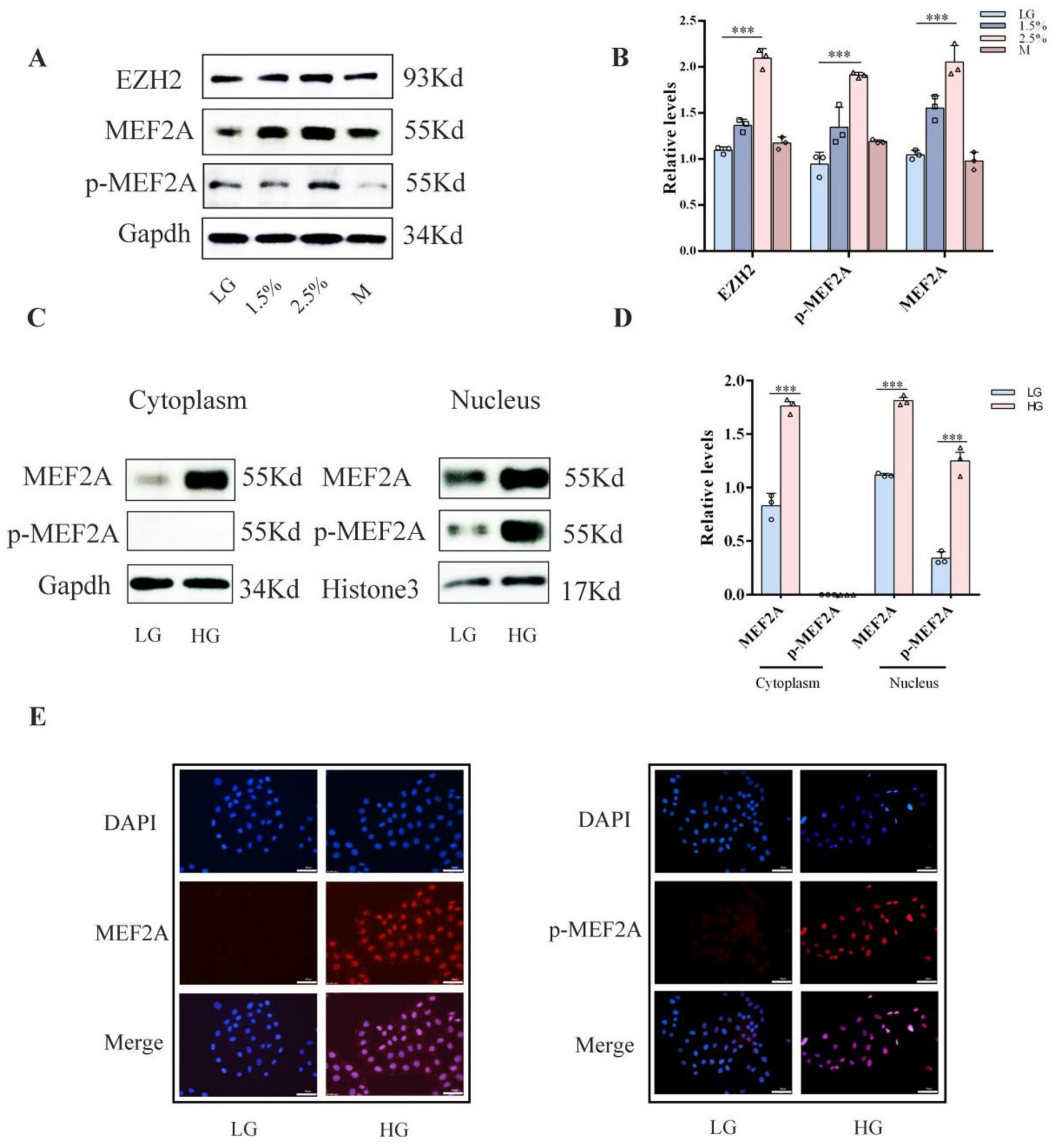
High glucose up-regulated the expression of MEF2A and p-MEF2A and promoted the nuclear translocation of p-MEF2A in HPMCs. (A) HPMCs were treated with a medium containing various concentrations of glucose (1.5%, 2.5%)and a medium containing 2.5% mannitol for 48h. Western blot analyses of EZH2, MEF2A and p-MEF2A in HPMCs under various treatments are shown. Gapdh served as a loading control. (B) Expression levels of EZH2, MEF2A and p-MEF2A were quantified by densitometry and normalized with Gapdh. (C) Western blot analysis of MEF2A and p-MEF2A expression levels in cytoplasm and nuclear proteins of HPMCs. (D) The relative expression of MEF2A and p-MEF2A. (E) Immunofluorescence analysis of MEF2A and p-MEF2A localization in HPMCs. MEF2A and p-MEF2A are stained red, nuclei are stained blue with DAPI (scale bar = 50 μm).

To investigate the effect of high glucose on the expression and phosphorylation of MEF2A, we treated HPMCs with high glucose. Western blot analysis revealed a robust upregulation in the protein levels of both total MEF2A and its phosphorylated form (p-MEF2A) in response to high glucose stimulation (Figure 1A, B). To elucidate the alteration in the subcellular localization of MEF2A under high glucose conditions, nuclear and cytoplasmic protein fractionation was performed on HPMCs stimulated with high glucose. Western blot and immunofluorescence analyses demonstrated that high glucose stimulation significantly increased the protein levels of MEF2A in both the cytoplasmic and nuclear compartments. Simultaneously, the expression of p-MEF2A was markedly upregulated and was predominantly concentrated in the nucleus (Figure 1C–E). Our data indicate that high glucose stimulation not only upregulates MEF2A and p-MEF2A expression in HPMCs but also leads to the nuclear accumulation of p-MEF2A.

### 3.2. Inhibition of MEF2A alleviates the high glucose-induced upregulation of EZH2 and EMT in HPMCs

**Figure 2.**
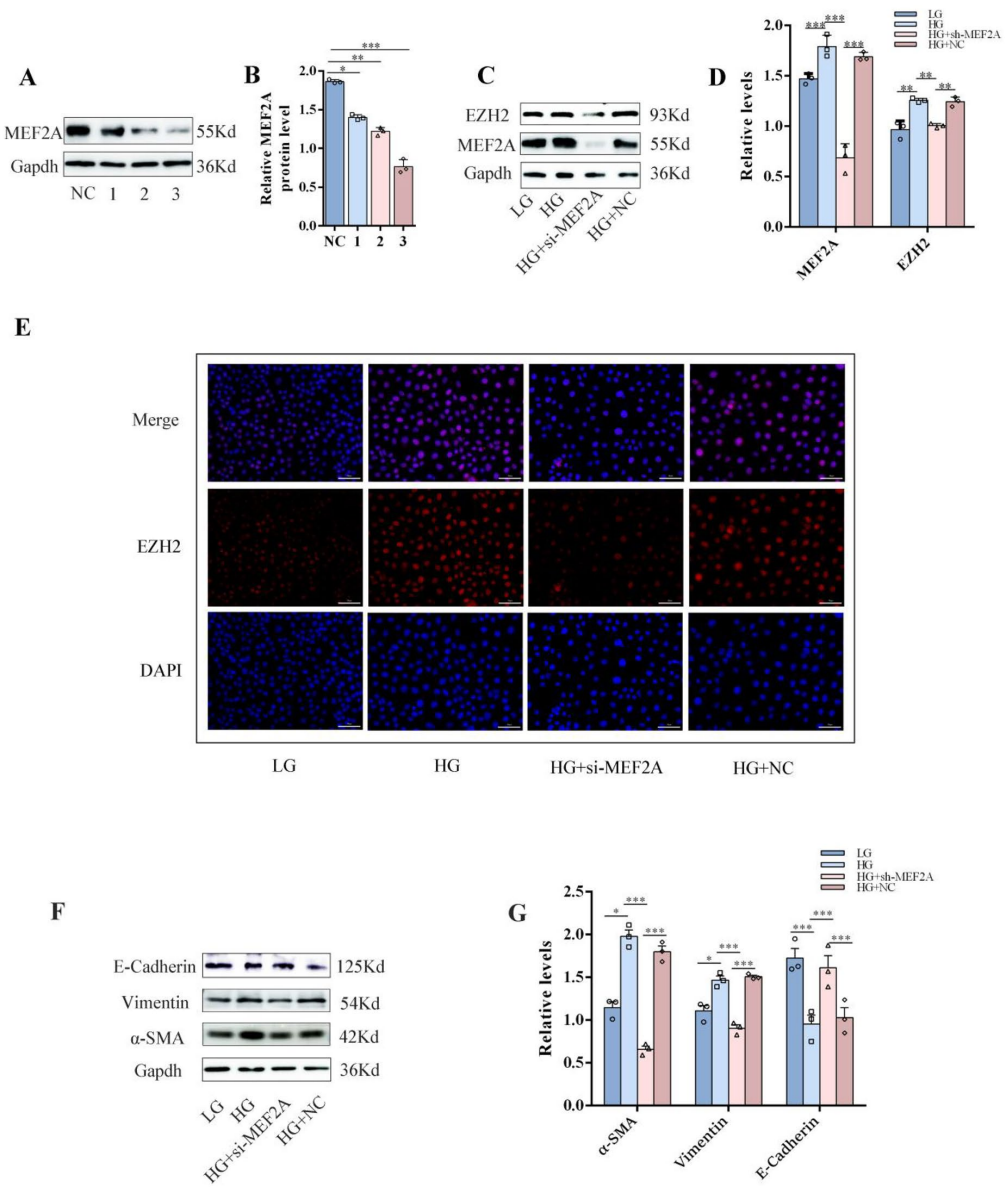
Inhibition of MEF2A alleviates the high glucose-induced upregulation of EZH2 and EMT in HPMCs. HPMCs were transfected with MEF2A siRNAs. (A)&(B) Identification of si-MEF2A efficiency in HPMCs. (C) Western blot assay of EZH2 and MEF2A in different groups. Gapdh served as a loading control. (D) The relative expression of EZH2 and MEF2A. (E) Immunofluorescence analysis of EZH2 in different groups. EZH2 is labeled in red (scale bar =50 μm). (Data are the mean ± S.D.; *p < 0.05, **p < 0.01, *** *p* < .001, *n* = 3.) (F) Western blot assay of E-Cadherin, α-SMA and Vimentin in different groups. Gapdh served as a loading control. (G) The relative expression of E-Cadherin, α-SMA and Vimentin.

To investigate the role of MEF2A in high glucose-induced EMT, we knocked down MEF2A expression in HPMCs using siRNA (Figure 2A, B). MEF2A knockdown significantly suppressed the high glucose-induced upregulation of EZH2 (Figure 2C–E) and effectively mitigated the EMT process. This was evidenced by the maintained expression of the epithelial marker E-cadherin and the marked downregulation of the mesenchymal markers α-SMA and Vimentin, as shown by Western blot analysis (Figure 2F, G).

### 3.3. Overexpression of EZH2 reversed the protective effect of si-MEF2A against high glucose-induced EMT in HPMCs

**Figure 3.**
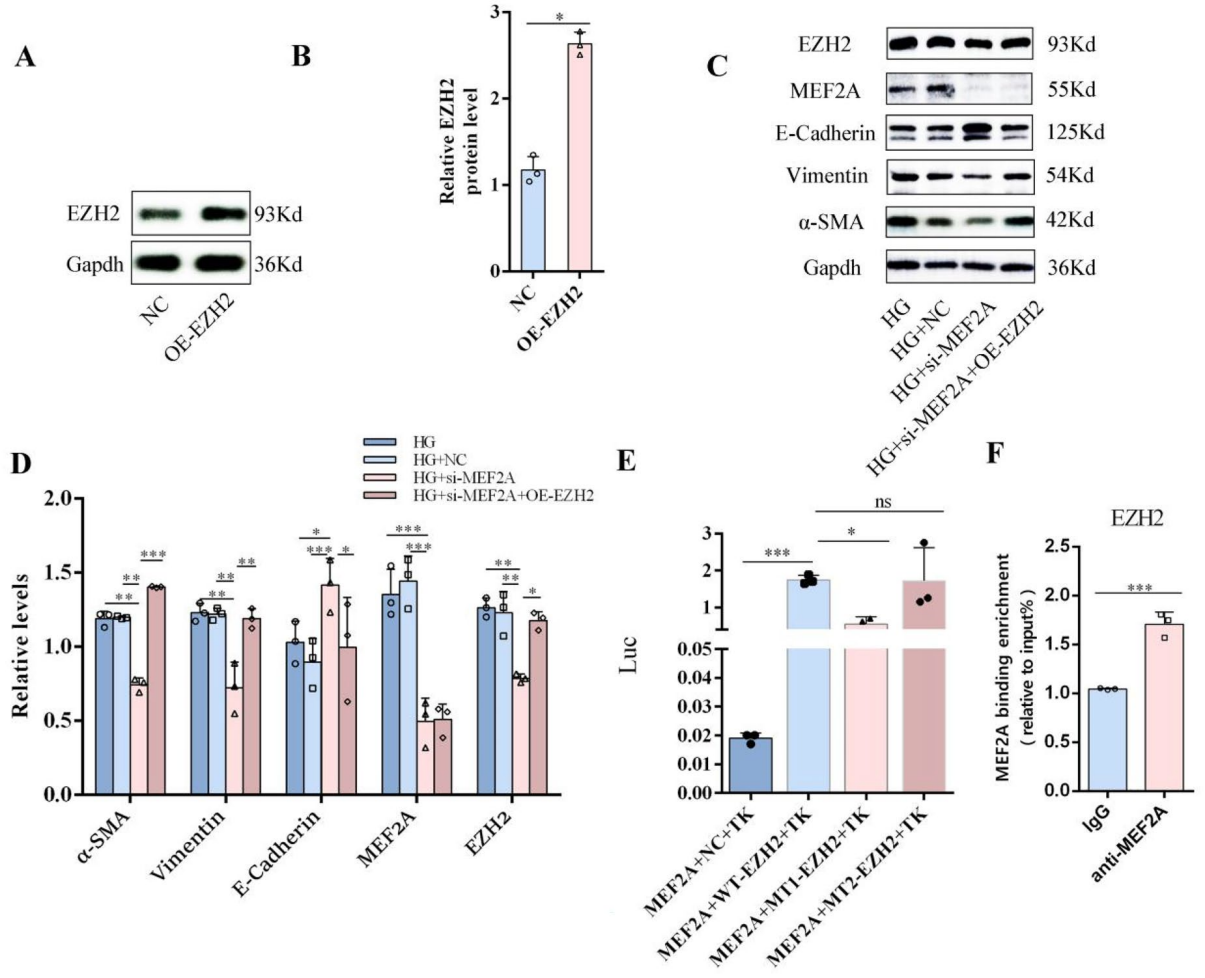
Overexpression of EZH2 reversed the protective effect of si-MEF2A against high glucose-induced EMT in HPMCs. Effect of EZH2 overexpression on HPMCs in the HG + si-MEF2A, HG + si-MEF2A + OE-EZH2, and HG + si-NC groups were transfected with si-MEF2A alone, si-MEF2A in combination with OE-EZH2, or si-NC, respectively. (A) Lentivirus was transfected into HPMCs to overexpress EZH2. The overexpression efficiency of EZH2 by lentivirus was detected by Western blot. Gapdh served as a loading control. (B) The relative expression of EZH2. (C) Western blot assay of EZH2, MEF2A, E-Cadherin, α-SMA and Vimentin in different groups. Gapdh served as a loading control. (D) The relative expression of EZH2, MEF2A, E-Cadherin, α-SMA and Vimentin. (Data are the mean ± S.D.; *p < 0.05, **p < 0.01,***p < 0.001, *n* = 3.) (E) Dual luciferase reporter assay of the regulatory effect of MEF2A on EZH2 promoter-driven luciferase activity in HPMCs. (Data are the mean ± S.D.; *p < 0.05, ***p < 0.001, *n* = 3.) (F) ChIP-qPCR showing the enrichment of MEF2A on promoter regions of EZH2. (Data are the mean ± S.D.; ***p < 0.001, *n* = 3).

To this end, we overexpressed EZH2 using a lentiviral system (Figure 3A, B) to assess its functional impact on the EMT process in HPMCs with MEF2A knockdown. Results indicate that EZH2 overexpression reverses the EMT process inhibited by MEF2A silencing by restoring the expression of α-SMA and vimentin and suppressing E-cadherin expression (Figure 3C, D). To investigate whether MEF2A directly transcriptionally regulates EZH2, we first designed two mutant EZH2 promoter plasmids (MT1 and MT2) using the NCBI and JASPAR databases. To this end, a dual-luciferase reporter assay was employed to assess the binding and transcriptional activity. Results from the dual-luciferase reporter assay indicated that MEF2A significantly enhanced the transcriptional activity of the EZH2 promoter. However, this enhancing effect was abolished when specific sites within the promoter were mutated (Figure 3E), suggesting that MEF2A transactivates EZH2 by directly binding to these putative response elements. To further investigate this interaction, MEF2A ChIP-qPCR was conducted. The results demonstrated significant enrichment of MEF2A at the EZH2 gene promoter (Figure 3F). These results provide evidence that MEF2A contributes to peritoneal fibrosis through the transcriptional activation of EZH2.

### 3.4. Suppression of the ROCK pathway reversed both the upregulation of MEF2A and the EMT process induced by high glucose in HPMCs

**Figure 4.**
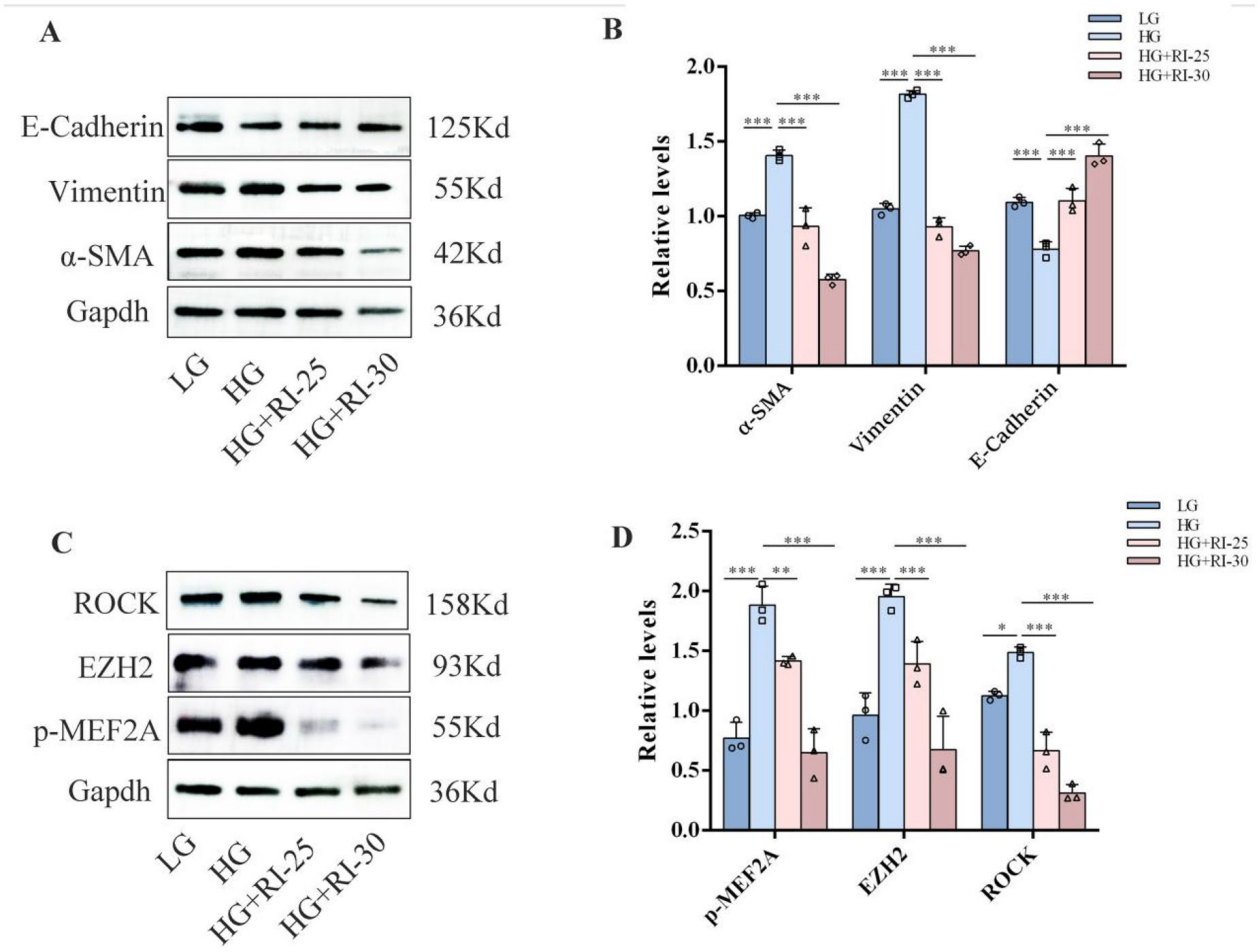
Suppression of the Rho/ROCK pathway reversed both the upregulation of MEF2A and the EMT process induced by high glucose in HPMCs. High glucose-stimulated HPMCs were treated with the ROCK-specific inhibitor Fasudil (25 μmol/L and 30 μmol/L). (A) Western blot assay of E-Cadherin, α-SMA and Vimentin. Gapdh served as a loading control. (B) The relative expression of E-Cadherin, α-SMA and Vimentin. (Data are the mean ± S.D.; *p < 0.05, **p < 0.01,***p < 0.001, *n* = 3.) (C) Western blot assay of ROCK, MEF2A, p-MEF2A in different groups. Gapdh served as a loading control. (D)The relative expression of ROCK, EZH2, MEF2A, p-MEF2A.(Data are the mean ± S.D.; *p < 0.05, **p < 0.01,***p < 0.001, *n* = 3).

In this study, high glucose–stimulated HPMCs were treated with the ROCK-specific inhibitor fasudil (Master of Bioactive Molecules, China). Firstly, fasudil was diluted with DMSO to prepare the storage solution, and then it was diluted with the culture medium to reach concentrations of 25 and 30 μmol/L respectively. Western blot analysis confirmed that inhibition of the ROCK pathway suppressed the high glucose-induced EMT process in HPMCs (Figure 4A, B). Further investigation revealed that ROCK pathway inhibition, achieved through fasudil treatment, concertedly suppressed the high glucose-induced upregulation of total MEF2A, its phosphorylated form (p-MEF2A), and EZH2 in total cellular proteins (Figure 4C, D).

### 3.5. Inhibition of the ROCK signaling pathway abolished high glucose-induced MEF2A phosphorylation and nuclear translocation

**Figure 5.**
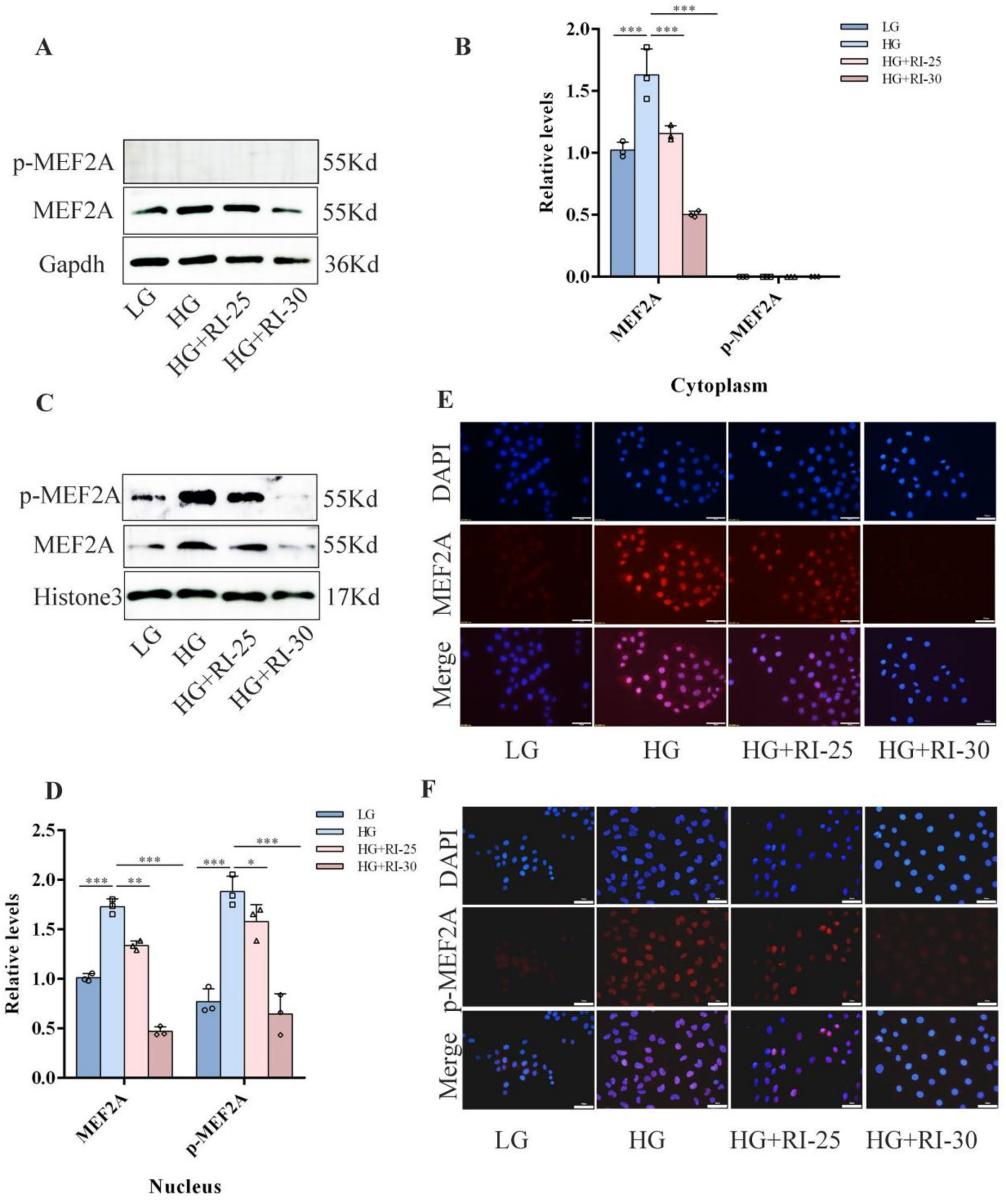
Inhibition of the Rho/ROCK signaling pathway abolished high glucose-induced MEF2A phosphorylation and nuclear translocation. (A) Western blot analysis of MEF2A and p-MEF2A expression levels in cytoplasm proteins of HPMCs. Gapdh served as a loading control. (B) The relative expression of MEF2A and p-MEF2A. (Data are the mean ± S.D.; *p < 0.05, **p < 0.01,***p < 0.001, *n* = 3.) (C) Western blot analysis of MEF2A and p-MEF2A expression levels in nuclear proteins of HPMCs. Histone3 served as a loading control. (D) The relative expression of MEF2A and p-MEF2A. (Data are the mean ± S.D.; *p < 0.05, **p < 0.01,***p < 0.001, *n* = 3.) (E)&(F) Immunofluorescence analysis of MEF2A and p-MEF2A localization in HPMCs. MEF2A and p-MEF2A are stained red, nuclei are stained blue with DAPI (scale bar =50 μm).

To investigate whether the RhoA/ROCK pathway regulates MEF2A subcellular localization under high glucose conditions, we performed nuclear and cytoplasmic protein fractionation in high glucose-stimulated HPMCs following treatment with the ROCK inhibitor Fasudil. Following fasudil treatment, Western blot analysis showed that MEF2A was redistributed, with reduced levels in the cytoplasm, while high glucose-induced nuclear accumulation of p-MEF2A was prevented (Figure 5A–D). This finding was further supported by immunofluorescence analysis (Figure 5E, F). This indicates that inhibiting the ROCK signaling pathway effectively alleviates the elevated protein expression and phosphorylation of MEF2A under high glucose, and also inhibits the nuclear translocation of p-MEF2A.

### 3.6. *In vivo* knockdown of MEF2A attenuates peritoneal dialysis-associated peritoneal fibrosis

**Figure 6.**
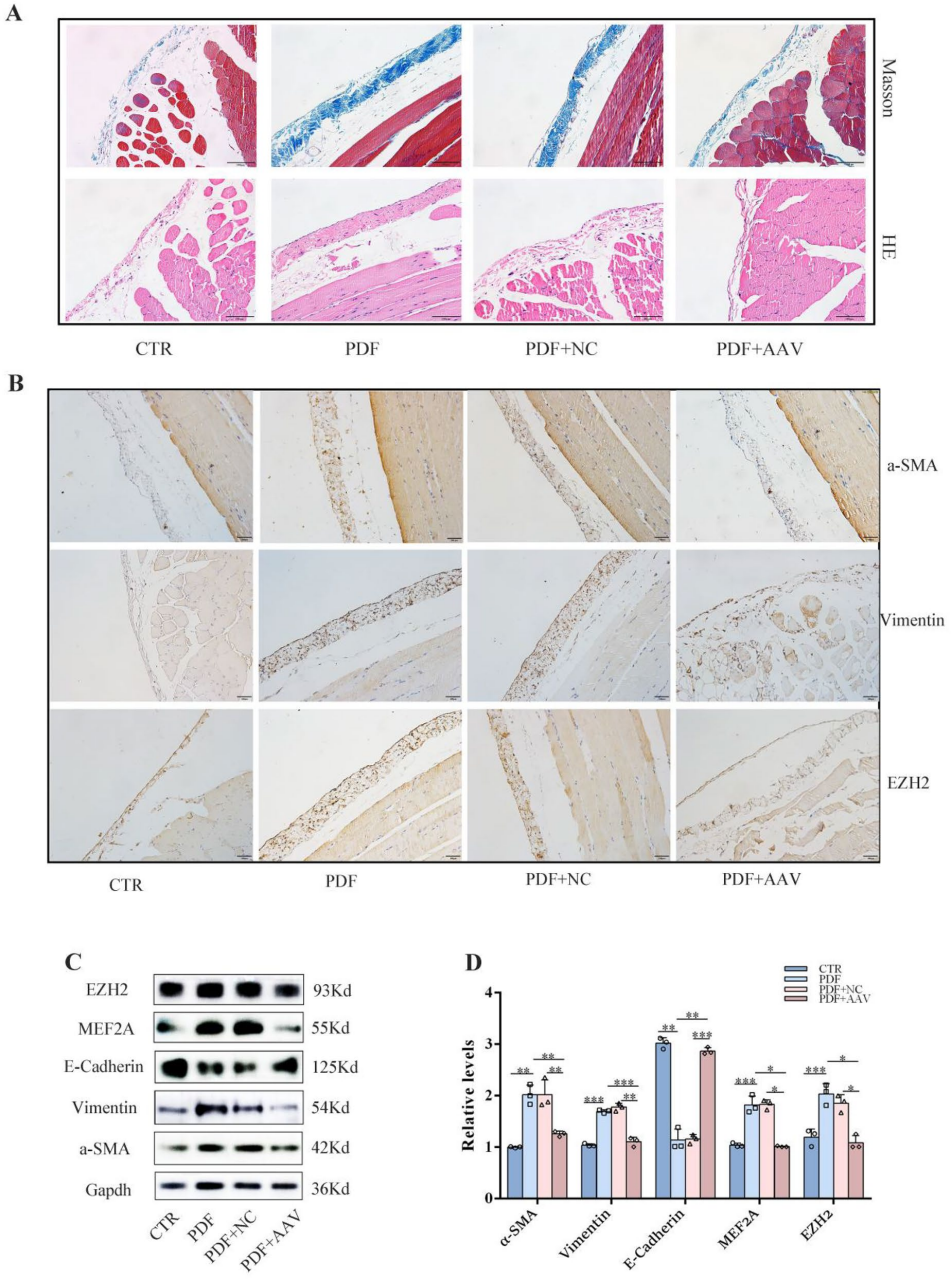
*In vivo* knockdown of MEF2A attenuates peritoneal dialysis-associated peritoneal fibrosis. (A) HE staining and Masson staining in peritoneal tissue of rat (scale =100μm). (B) Immunohistochemical analysis of α-SMA, Vimentin, and EZH2 in peritoneal tissue of rat (scale =100μm). (C) Western blot analyses of EZH2, MEF2A, E-Cadherin, α-SMA and Vimentin in different groups. Gapdh served as a loading control. (D) The relative expression of EZH2, MEF2A, E-Cadherin, α-SMA and Vimentin. (Data are the mean ± S.D.; *p < 0.05, **p < 0.01, ***p < 0.001, *n* = 3).

To confirm the effect of MEF2A on PD-associated PF, we silenced MEF2A in SD rats *via* intraperitoneal injection of AAV. HE and Masson staining revealed that MEF2A knockdown significantly attenuated peritoneal thickness and reduced collagen deposition in rat peritoneal tissues (Figure 6A). Immunohistochemistry and Western blot results demonstrated that inhibition of MEF2A prevented the downregulation of E-cadherin expression in peritoneal tissue, while the expression of mesenchymal markers α-SMA and Vimentin was significantly suppressed. Concurrently, EZH2 expression was also reduced (Figure 6B, C, D).

### 3.7. Inhibition of the ROCK pathway suppresses peritoneal dialysis-associated peritoneal fibrosis and MEF2A overexpression *in vivo*

**Figure 7.**
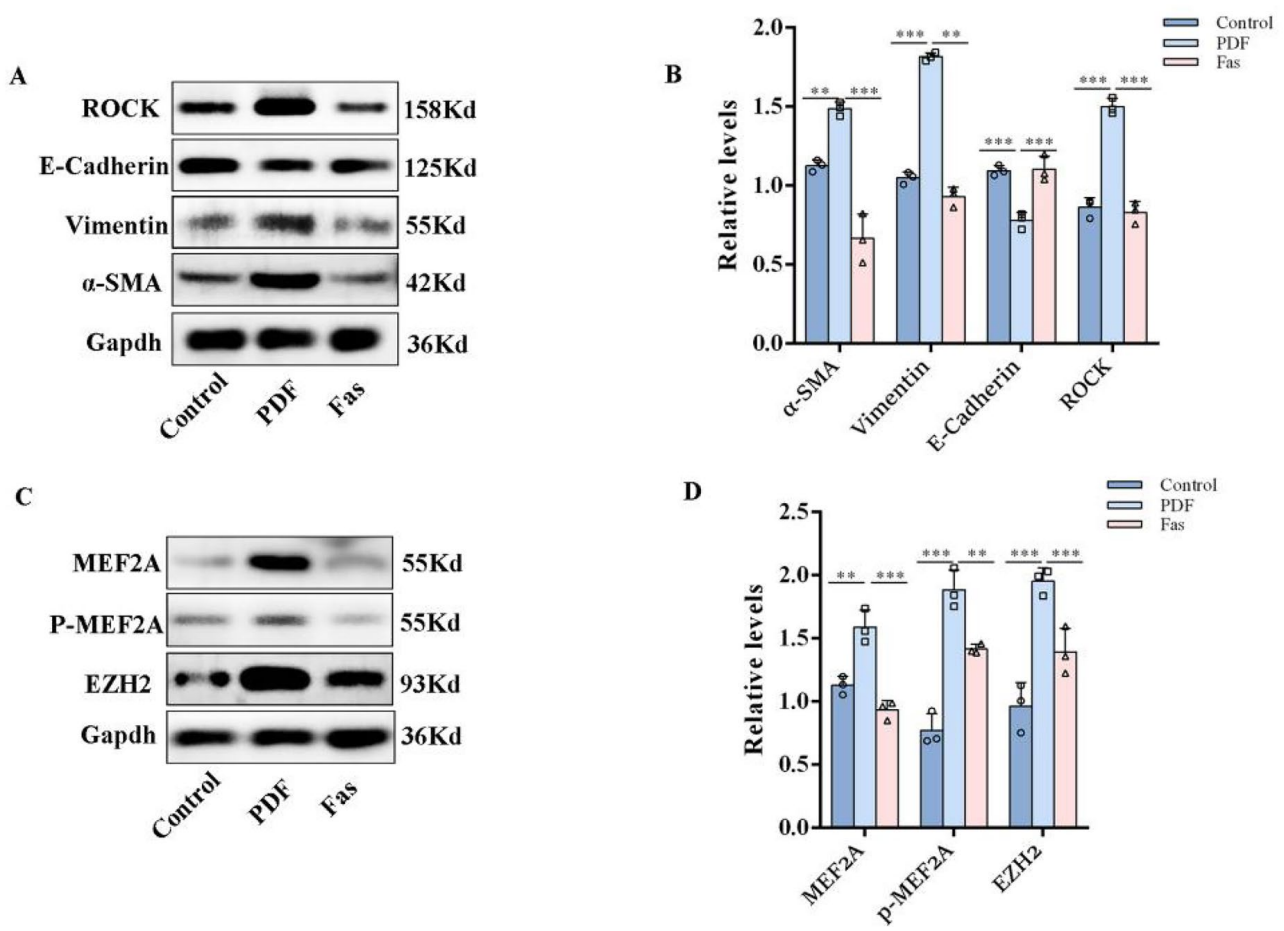
Inhibition of the Rho/ROCK pathway suppresses peritoneal dialysis-associated peritoneal fibrosis and MEF2A overexpression *in vivo*. (A) Western blot assay of ROCK, E-Cadherin, α-SMA and Vimentin in different groups. Gapdh served as a loading control. (B) The relative expression of ROCK, E-Cadherin, α-SMA and Vimentin. (Data are the mean ± S.D.; *p < 0.05, **p < 0.01, ***p < 0.001, *n* = 3.) (C) Western blot assay of ROCK, EZH2, MEF2A and p-MEF2A in different groups. Gapdh served as a loading control. (D) The relative expression of EZH2, MEF2A and p-MEF2A. (Data are the mean ± S.D.; *p < 0.05, **p < 0.01, ***p < 0.001, *n* = 3).

To determine whether ROCK pathway inhibition affects MEF2A expression in peritoneal fibrosis, rats were treated with fasudil *via* oral gavage. As shown by Western blot, fasudil treatment attenuated the EMT (Figure 7A, B) and significantly suppressed the associated upregulation of MEF2A and EZH2 in fibrotic peritoneal tissues (Figure 7C, D).

## 4. Disscussion

Long-term peritoneal dialysis is a life-sustaining treatment for end-stage kidney disease, but this treatment can lead to the gradual development of irreversible peritoneal fibrosis. Its characteristics include excessive deposition of extracellular matrix proteins, thickening of the peritoneal membrane, and loss of its functional integrity. Although PD holds significant clinical importance, the underlying mechanisms leading to the development of peritoneal fibrosis remain unclear. The pathogenesis of PF is multifactorial, involving chronic inflammation, oxidative stress, mechanical stimulation from dialysate, and dysregulation of key signaling pathways [3,4,31]. Our research revealed previously unrecognized interactions among these pathways and provided new potential therapeutic targets for alleviating peritoneal fibrosis and improving the prognosis of peritoneal dialysis patients.

Most experiments have demonstrated that the ROCK is indeed involved in the process of organ fibrosis [17,32]. There are two isoforms of ROCK, ROCK 1 and ROCK 2, and ROCK 1/2 inhibition has been shown to ameliorate lung, heart, kidney, and liver fibrosis. Numerous studies have also shown that the intensity of ROCK activation is strongly positively correlated with the amount of collagen deposition, and inhibiting the ROCK pathway can significantly reduce the fibrotic area in various organs. Studies have shown that in the liver, ROCK2 selectively regulates the mitochondrial dysfunction of hepatic stellate cells, inducing the collapse of mitochondrial membrane potential. In lung tissue, ROCK1 is shown to be the main driver of EMT, promoting the loss of E-cadherin. In the case of kidney-specificity, ROCK causes the phosphorylation of nephrin-Y1217, thereby disrupting the podocyte cytoskeleton and leading to the occurrence of proteinuria [16,33,34]. Thus, ROCK plays a key role in the development of fibrosis in most organs. There are also many studies that have demonstrated that inhibiting the ROCK pathway ameliorates peritoneal fibrosis. In this study, we applied, a novel inhibitor of Rho kinase, to inhibit signaling in the ROCK signaling pathway [35]. Some studies have shown that Fasudil can reduce or even block fibrosis in the heart, kidney and other organs [36–39]. Therefore, we speculate that inhibiting the ROCK pathway may also help to slow down the progression of peritoneal fibrosis. In this study, we applied Fasudil to suppress EMT in peritoneal mesothelial cells after long periods with high glucose concentrations. We found that after the inhibition of ROCK pathway, the expression of EMT-related indicators was significantly inhibited, including the expression of MEF2A. Subsequently, we speculated that MEF2A is a signaling molecule downstream of the ROCK pathway. We later confirmed this speculation by isolating the cytosol and exonuclear proteins and analyzing the expression of MEF2A and p-MEF2A, and we found that the expression of phosphorylated MEF2A into the nucleus was inhibited after Fasudil addition, and this finding was also confirmed by immunofluorescence.

MEF2A belongs to a member of the myocyte enhancer-2 family and is a well-established transcription factor [20]. Many studies have found that MEF2A plays an important role in the occurrence and progression of many tumors, such as prostate cancer and breast cancer [22,40]. Moreover, MEF2A was also found to promote fibroblast activation and skin fibrosis by mediating the silencing of SMAD7 [21]. And MEF2A was also found to bind to the promoter region and initiate ZEB2 transcription to promote EMT progression, thereby promoting fibrosis of the tissue [41]. Studies have shown that the role of MEF2A in cardiac development, skeletal muscle differentiation, and neuronal survival has been widely reported, but its function in fibrosis, especially in kidney and peritoneal-related fibrosis diseases, has not been deeply explored. Some studies have found that EZH2 plays an important role in the progression of peritoneal fibrosis [30,42], which is also reflected in our previous studies. We speculate that there may be some link between MEF2A and EZH2. We designed a small interfering RNA of MEF2A to silence MEF2A expression and found that, not only the expression of EMT-related indicators but also EZH2 expression was significantly suppressed after silencing of MEF2A.Therefore, we speculate that EZH2 is downstream of MEF2A, and this was confirmed by a series of immunofluorescence, etc.

**Figure 8.**
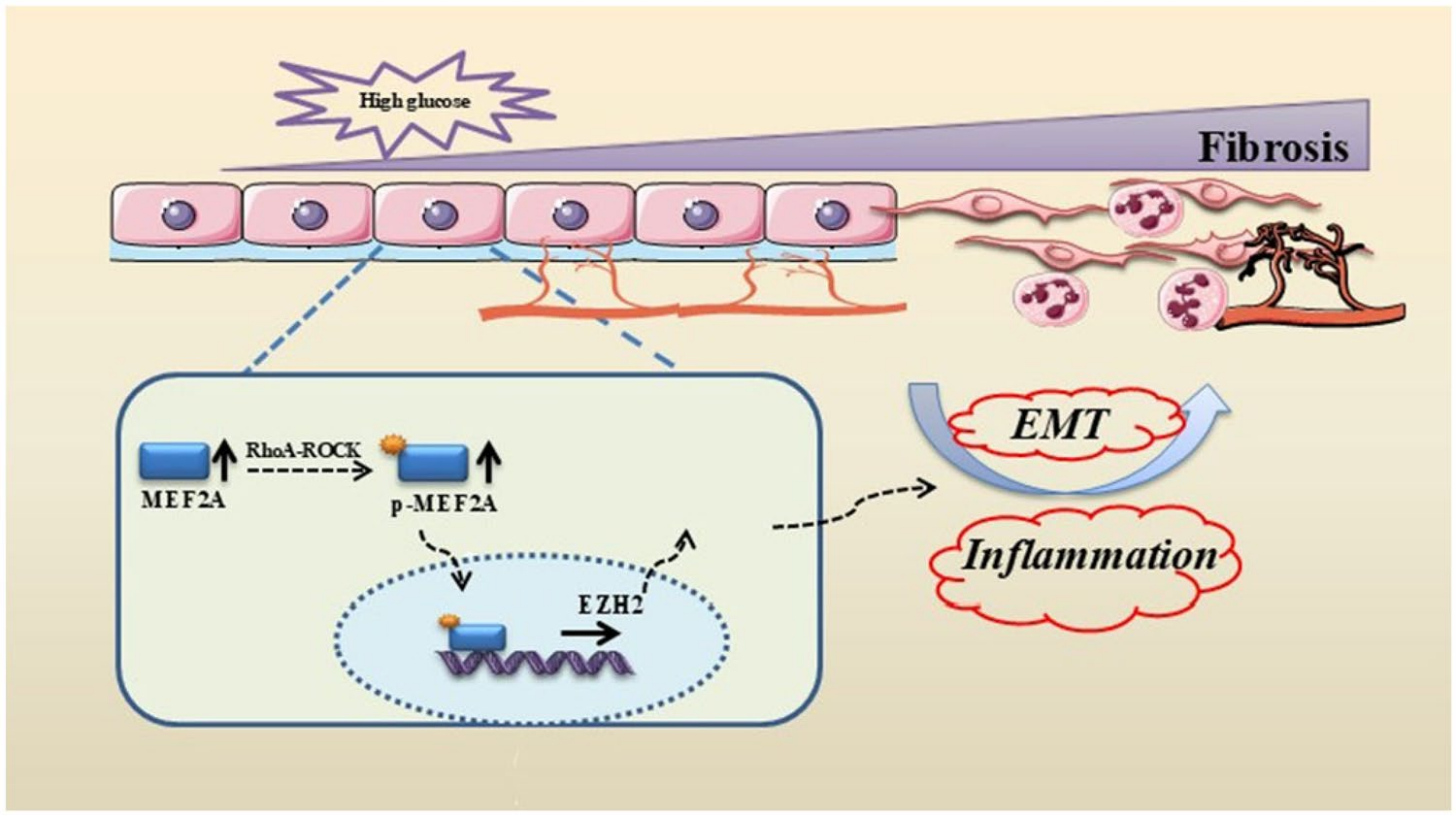
The mechanism of the ROCK/MEF2A/EZH2 signaling axis in peritoneal fibrosis. MEF2A nuclear translocation mediated by ROCK phosphorylation promotes EZH2 expression and peritoneal fibrosis.

In conclusion, to summarize the key findings of this study, we have constructed a schematic model (Figure 8). It delineates a novel ROCK/MEF2A/EZH2 signaling axis in the pathogenesis of peritoneal fibrosis associated with long-term dialysis. Specifically, high-glucose conditions activate the ROCK pathway, which leads to the upregulation and nuclear translocation of MEF2A. MEF2A, in turn, directly binds to and transcriptionally activates EZH2, thereby driving epithelial-mesenchymal transition (EMT) and fibrosis. This provides a new insight into peritoneal fibrosis pathogenesis and highlights MEF2A as a promising therapeutic target for delaying peritoneal fibrosis.

## Data Availability

The datasets used and/or analyzed during the current study are available from the corresponding author on reasonable request.
